# Early predictors of delayed radiographic resolution of lobar pneumonia caused by *Mycoplasma pneumoniae* in children: a retrospective study in China

**DOI:** 10.1186/s12879-024-09289-x

**Published:** 2024-04-19

**Authors:** Yu Zheng, Guoshun Mao, Hongchen Dai, Guitao Li, Liying Liu, Xiaying Chen, Ying Zhu

**Affiliations:** Department of Pediatrics, Fu Yang People’s Hospital, No.501, Sanqing Road, Yingzhou District, 236000 Fuyang, Anhui Province China

**Keywords:** Children, *Mycoplasma pneumoniae*, Lobar pneumonia, Imaging

## Abstract

**Background:**

Lobar pneumonia caused by *Mycoplasma pneumoniae* is a relatively difficult-to-treat pneumonia in children. The time of radiographic resolution after treatment is variable, a long recovery time can result in several negative effects, and it has attracted our attention. Therefore, exploring factors associated with delayed radiographic resolution will help to identify these children at an early stage and prepare for early intervention.

**Methods:**

The data of 339 children with lobar pneumonia caused by *Mycoplasma pneumoniae* were collected from the Department of Pediatrics of Fu Yang People’s Hospital, China from January 2021 to June 2022. After discharge, the children were regularly followed up in the outpatient department and on the WeChat platform for > 8 weeks. According to whether pulmonary imaging (chest radiography or plain chest computed tomography) returned to normal within 8 weeks, the children were divided into the delayed recovery group (DRG) (*n* = 69) and the normal recovery group (NRG) (*n* = 270). The children’s general information, laboratory examination findings, bronchoscopy results, and imaging findings were retrospectively analyzed. Single-factor analysis was performed to identify the risk factors for delayed radiographic resolution of lobar pneumonia caused by *Mycoplasma pneumoniae*, and the factors with statistically significant differences underwent multiple-factor logistic regression analysis. Receiver operating characteristic (ROC) analysis was then performed to calculate the cutoff value of early predictive indicators of delayed radiographic resolution.

**Results:**

Single-factor analysis showed that the following were significantly greater in the DRG than NRG: total fever duration, the hospitalization time, C-reactive protein (CRP) level, lactate dehydrogenase (LDH) level, D-dimer level, pulmonary lesions involving two or more lobes, a large amount of pleural effusion, the time to interventional bronchoscopy, and mucus plugs formation. Multivariate logistic regression analysis showed that the hospitalization time, CRP level, LDH level, pulmonary lesions involving two or more lobes, and a large amount of pleural effusion were independent risk factors for delayed radiographic resolution of lobar pneumonia caused by *Mycoplasma pneumoniae*. The cutoff values on the receiver operating characteristic curve were a hospitalization time of ≥ 10.5 days, CRP level of ≥ 25.92 mg/L, and LDH level of ≥ 378 U/L.

**Conclusion:**

If patients with lobar pneumonia caused by *Mycoplasma pneumoniae* have a hospitalization time of ≥ 10.5 days, CRP level of ≥ 25.92 mg/L, and LDH level ≥ 378 U/L, the time of radiographic resolution is highly likely to exceed 8 weeks. Pediatricians must maintain a high level of vigilance for these factors, control the infection as early as possible, strengthen airway management, and follow up closely to avoid complications and sequelae of *Mycoplasma pneumoniae* pneumonia.

## Introduction

Lobar pneumonia is a common type of *Mycoplasma pneumoniae* pneumonia (MPP) in children, and it is typically a mild condition that responds well to macrolide antibiotics (MAs). A follow-up study revealed that in approximately 70% of children’s pulmonary imaging changes can return to normal within 8 weeks when antibiotics and glucocorticoids are used appropriately [1, 2]. In some children, however, the time of radiographic resolution significantly exceeds 8 weeks. When the clinical condition is accompanied by necrotizing pneumonia, the time of radiographic resolution can reach 6 months [3], significantly impacting the patient’s quality of life. Furthermore, children with long-term lung lesions may develop persistent fever, impaired lung function, and worsening of the disease [4, 5].

This study was performed to identify predictors that can be used for early prediction of delayed radiographic resolution of lobar pneumonia caused by *Mycoplasma pneumoniae* (*M. pneumoniae*) in children, thus facilitating early interventions to prevent serious complications such as necrotizing pneumonia, bronchiectasis, and bronchial occlusion.

## Patients and methods

### Patients

**Fig. 1:**
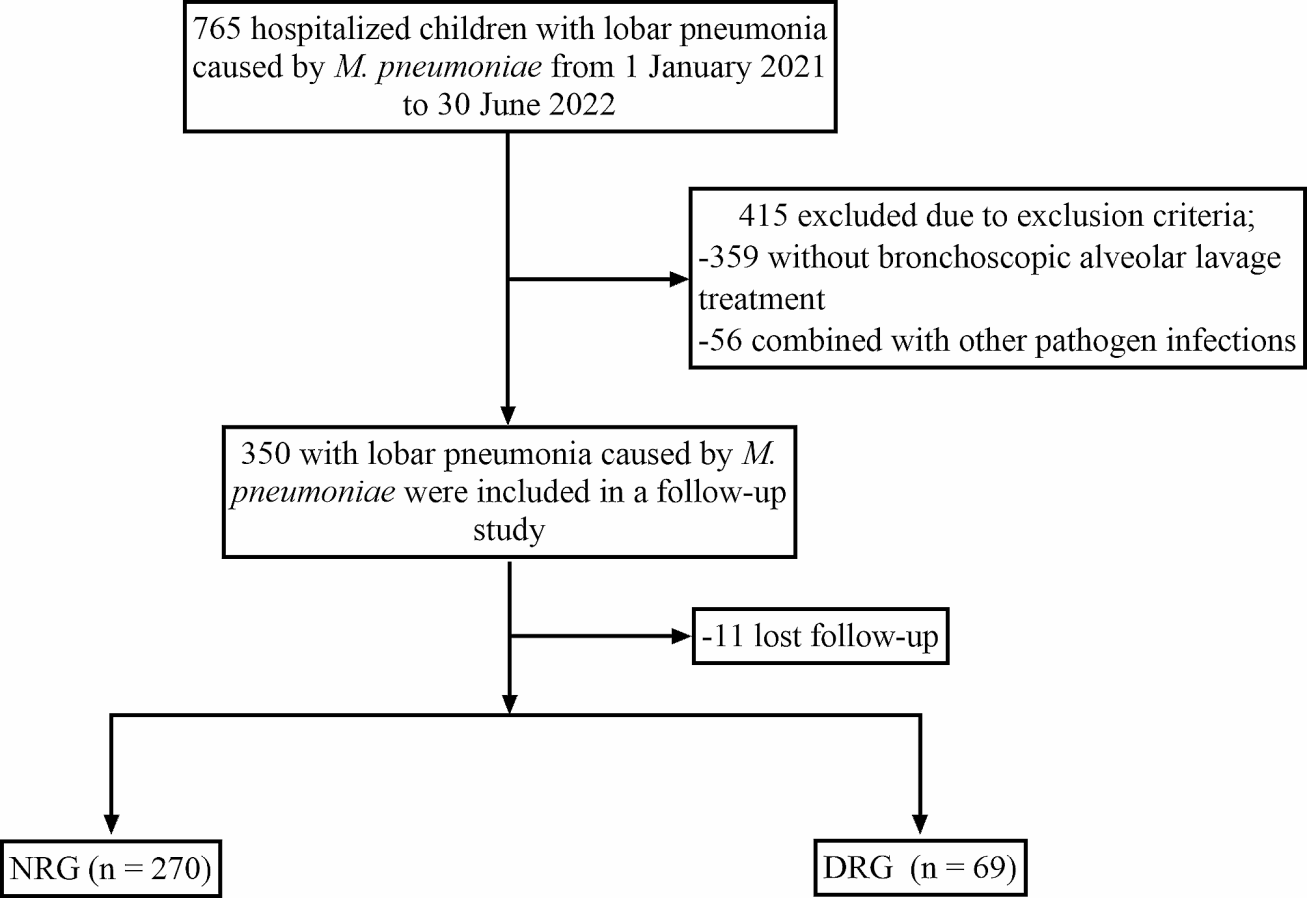
Flow chart of patient selection

We retrospectively analyzed the data of 339 children with lobar pneumonia caused by *M. pneumoniae* who were admitted to the Department of Pediatrics of Fu Yang People’s Hospital from 1 January 2021 to 30 June 2022. The children were divided into a normal recovery group (NRG) (*n* = 270) and a delayed recovery group (DRG) (*n* = 69) (**Fig. 1**). The research protocol was approved by the Ethical Committee of Fu Yang People’s Hospital (ethical code: 2018 − 167).

### Inclusion and exclusion criteria

The inclusion criteria were (1) chest radiography or lung computed tomography findings consistent with the imaging characteristics of lobar pneumonia and (2) satisfaction of the diagnostic criteria for MPP in the Expert Consensus on Laboratory Diagnostics and Clinical Practice of Mycoplasma pneumoniae Infection in Children in China (i.e., manifestations of pneumonia and/or imaging changes combined with an MP antibody titer of ≥ 1:160) [6].

The exclusion criteria were (1) infection with other pathogens, (2) immunodeficiency, (3) lack of bronchoscopy with bronchoalveolar lavage, (4) incomplete medical records, and (5) loss to follow-up.

### Data collection

The following data were retrospectively collected from the children’s medical records. (1) Clinical data: age, sex, total fever duration, peak body temperature, hospitalization time, season of onset, and time to interventional bronchoscopy ( The “time” refers to the interval from the onset of illness to undergoing bronchoscopy with bronchoalveolar lavage, it is not a treatment time.). (2) Laboratory examination findings: including white blood cell count (WBC), neutrophil ratio (NE%), lymphocyte ratio (LYM%), C-reactive protein (CRP) level, D-dimer level, lactate dehydrogenase (LDH) level, alkaline phosphatase (ALP) level, and immunoglobulins A, M, and G (IgA, IgM, IgG). (3) Other conditions: glucocorticoid resistance, a large amount of pleural effusion, atelectasis, lung lesions involving two or more lobes, and mucus plugs formation as shown by bronchoscopy. A retrospective analysis was performed to understand the relationship between the above factors and delayed radiographic resolution of lobar pneumonia caused by *M. pneumoniae* in children.

### Definitions

(1) According to previous reports, the time of radiographic resolution in most patients with lobar pneumonia caused by *M. pneumoniae* is 1 to 2 months [7–9]. Therefore, we defined delayed radiographic resolution as lack of recovery on chest radiographs or lung computed tomography scans from admission to 8 weeks of treatment. Radiologic follow-up was performed every 4 weeks.

(2) MAs non-responsiveness was defined as a persistent fever in a child with MPP after 72 h of regular MAs treatment, with either no improvement in clinical signs and pulmonary imaging findings or the development of further aggravation [10].

(3) Glucocorticoid resistance was defined as a persistent fever for > 72 h after intravenous administration of methylprednisolone at 1 to 2 mg/kg per day [11, 12].

(4) A large amount of pleural effusion was defined as a total pleural fluid volume of ≥ 500 mL and a low to medium volume of pleural effusion was defined as a total pleural fluid volume of < 500 mL.

### Bronchoscopy with bronchoalveolar lavage

All children in this study received routine anti-infection therapy and nebulization for 3 days after admission. If the children still had persistent fever, persistent cough, and no significant improvement in pulmonary signs, bronchoscopy with bronchoalveolar lavage was recommended. The alveolar lavage fluid was reserved for pathogenic nucleic acid detection after obtaining written informed consent from the child’s family. The equipment used for bronchoscopy with alveolar lavage is PENTAX fiber bronchoscopy (device model: EB-1170 K).

Patients were discharged when the rales disappeared, and the body temperature was normal for ≥ 3 consecutive days after completing routine anti-infective and anti-inflammatory treatment and bronchoscopy with bronchoalveolar lavage.

### Statistical analysis

The data were analyzed using SPSS 25.0 software (IBM Corp., Armonk, NY, USA). Measurement data conforming to a normal distribution are presented as mean ± standard deviation, and data with a skewed distribution are presented as median with interquartile range (Q25–Q75). Comparisons between groups were performed with the t-test or Mann–Whitney U test [13]. Enumerated data are expressed as rates, and the χ^2^ test was used for comparison between groups. We conducted a single-factor analysis to identify the risk factors for delayed radiographic resolution. We then conducted a multivariate logistic regression analysis of statistically significant factors to summarize the high-risk factors. Finally, the relevant factors were analyzed using a receiver operating characteristics (ROC) curve, and the early predictors of delayed radiographic resolution were summarized. Two-sided *P*˗values of < 0.05 were considered statistically significant.

## Results

### Clinical features

#### General information

A total 339 children with lobar pneumonia caused by *M. pneumoniae* were divided into 270 cases with NRG and 69 cases with DRG. The mean age at onset was 7.17 ± 2.37 years in the NRG and 6.86 ± 1.50 years in the DRG. The male: female ratio was approximately 1.4:1.0 (NRG vs. DRG). There was no significant difference in sex, season of onset, or age between the two groups. Total fever duration and hospitalization time were significantly longer in the DRG than NRG (*P* < 0.05). The time to interventional bronchoscopy was significantly longer in the DRG than NRG (11.06 ± 3.53 vs. 9.03 ± 2.76 days, respectively) (Table 1).

**Table 1:** Comparison of clinical data between NRG and DRG

Characteristics	NRG (*n* = 270)	DRG (*n* = 69)	*P*˗value
Gender (male/female)	159/111	36/33	0.098
Onset seasons (spring and summer/autumn and winter)	138/132	29/40	0.178
Age (years)	7.17 ± 2.37	6.86 ± 1.50	0.179
Total fever duration (days)	6.34 ± 2.63	11.13 ± 4.37	< 0.001
Peak body temperature (℃)	39.15 ± 0.73	39.29 ± 0.64	0.114
Hospitalization time (days)	8.36 ± 2.26	12.70 ± 4.07	< 0.001
Time to interventional bronchoscopy (days)	9.03 ± 2.76	11.06 ± 3.53	< 0.001

NRG, normal recovery group; DRG, delayed recovery group.

### Laboratory test results

Comparison of the laboratory examination results between the two groups showed that the CRP, D˗dimer, and LDH levels were significantly higher in the DRG than NRG (*P* < 0.05). There were no significant differences in the white blood cell count, neutrophil ratio, lymphocyte ratio, alkaline phosphatase level, or immunoglobulin levels (*P* > 0.05) (Table 2).

**Table 2:** Comparison of laboratory examination results between NRG and DRG

Characteristics	NRG (*n* = 270)	DRG (*n* = 69)	*P*˗value
WBC (×10^9^/L)	7.27 ± 2.24	7.72 ± 1.75	0.121
NE%	61.74 ± 11.14	64.79 ± 12.68	0.068
LYM%	29.20 ± 9.55	27.47 ± 10.82	0.223
CRP (mg/L)	10.61(5.21, 17.03)	57.18(35.11, 98.09)	< 0.001
D-D (mg/L)	0.85(0.41, 2.21)	3.18(2.09, 5.53)	< 0.001
ALP (U/L)	174.22 ± 52.42	163.12 ± 37.31	0.099
LDH (U/L)	307.99 ± 58.77	515.00 ± 215.48	< 0.001
IgA (g/L)	1.37 ± 0.55	1.45 ± 0.70	0.418
IgM (g/L)	1.47 ± 0.52	1.55 ± 0.74	0.251
IgG (g/L)	8.85 ± 1.88	9.00 ± 1.97	0.569

NRG, normal recovery group; DRG, delayed recovery group; WBC, white blood cell count; NE%, neutrophil ratio; LYM%, lymphocyte ratio; CRP, C-reactive protein; D-D, D-dimer; LDH, lactate dehydrogenase; ALP, alkaline phosphatase; IgA, immunoglobulin A; IgM, immunoglobulin M; IgG, immunoglobulin G

### Other conditions

There were statistically significant differences in glucocorticoid resistance, a large amount of pleural effusion, atelectasis, pulmonary lesions involving two or more lung lobes, and mucus plugs formation between the NRG and DRG (*P* < 0.05). However, the incidence of MAs non-responsiveness was not significantly different between the two groups (Table 3).

**Table 3:** Comparison of other conditions between NRG and DRG

Characteristics	NRG (*n* = 270)	DRG (*n* = 69)	*P*˗value
MAs non-responsiveness [*n*(%)]	105(38.9)	35(50.7)	0.075
Glucocorticoid resistance [*n*(%)]	27(10.0)	40(58.0)	< 0.001
A large amount of pleural effusion [*n*(%)]	20(7.4)	28(40.6)	< 0.001
Atelectasis [*n*(%)]	54(20.0)	29(42.0)	< 0.001
Pulmonary lesions involving two or more lung lobes [*n*(%)]	54(20.0)	50(72.5)	< 0.001
Mucus plugs [*n*(%)]	59(21.9)	38(55.1)	< 0.001

NRG, normal recovery group; DRG, delayed recovery group; MAs, macrolide antibiotics

### Multivariate logistic regression analysis

The statistically significant factors in the above-described univariate analysis were taken as independent variables, and whether delayed recovery had occurred was taken as the dependent variable (no = 0, yes = 1); a binary logistic regression analysis was then performed. The final results showed that the hospitalization time [odds ratio (OR) = 1.584, 95% confidence interval (CI) = 1.082–2.321, *P* = 0.018], LDH level (OR = 1.022, 95% CI = 1.008–1.036, *P* = 0.002), CRP level (OR = 1.079, 95% CI = 1.045–1.114, *P* < 0.001), pulmonary lesions involving two or more lung lobes (OR = 8.997, 95% CI = 1.698–47.682, *P* = 0.010), and a large amount of pleural effusion (OR = 11.568, 95% CI = 1.767–75.728, *P* = 0.011) were independent risk factors for delayed radiographic resolution of lobar pneumonia caused by *M. pneumoniae* in children (*P* < 0.05) (Table 4).

**Table 4:** Multivariate logistic regression analysis of delayed radiographic resolution of lobar pneumonia caused by Mycoplasma pneumoniae in children

Variables	B	SE	Wald	*P*-value	Odds ratio 95% confidence interval (CI)
Hospitalization time	0.460	0.195	5.583	0.018	1.584(1.082–2.321)
LDH	0.022	0.007	9.648	0.002	1.022(1.008–1.036)
CRP	0.076	0.016	21.734	< 0.001	1.079(1.045–1.114)
Pulmonary lesions involving two or more lung lobes	2.197	0.851	6.666	0.010	8.997(1.698–47.682)
A large amount of pleural effusion	2.448	0.959	6.522	0.011	11.568(1.767–75.728)
Total fever duration	0.183	0.157	1.369	0.242	1.201(0.884–1.632)
Glucocorticoid resistance	1.476	0.917	2.592	0.107	4.375(0.725–26.381)
D-D	-0.180	0.101	3.154	0.076	0.835(0.685–1.019)
Mucus plugs	-0.686	0.874	0.616	0.432	0.503(0.091–2.793)
Time to interventional bronchoscopy	0.128	0.105	1.484	0.223	1.137(0.925–1.398)
Atelectasis	0.902	0.831	1.179	0.278	2.465(0.484–12.560)

Hospitalization time, CRP level, LDH level, D-D level, total fever duration and time to interventional bronchoscopy are measurement data. Pulmonary lesions involving two or more lung lobes, a large amount of pleural effusion, glucocorticoid resistance, mucus plugs and atelectasis are enumerated data (0 = no, 1 = yes)

NRG, normal recovery group; DRG, delayed recovery group; LDH, lactate dehydrogenase; CRP, C-reactive protein; D-D, D-dimer; SE, standard error

### ROC curve regression analysis

**Fig. 2:**
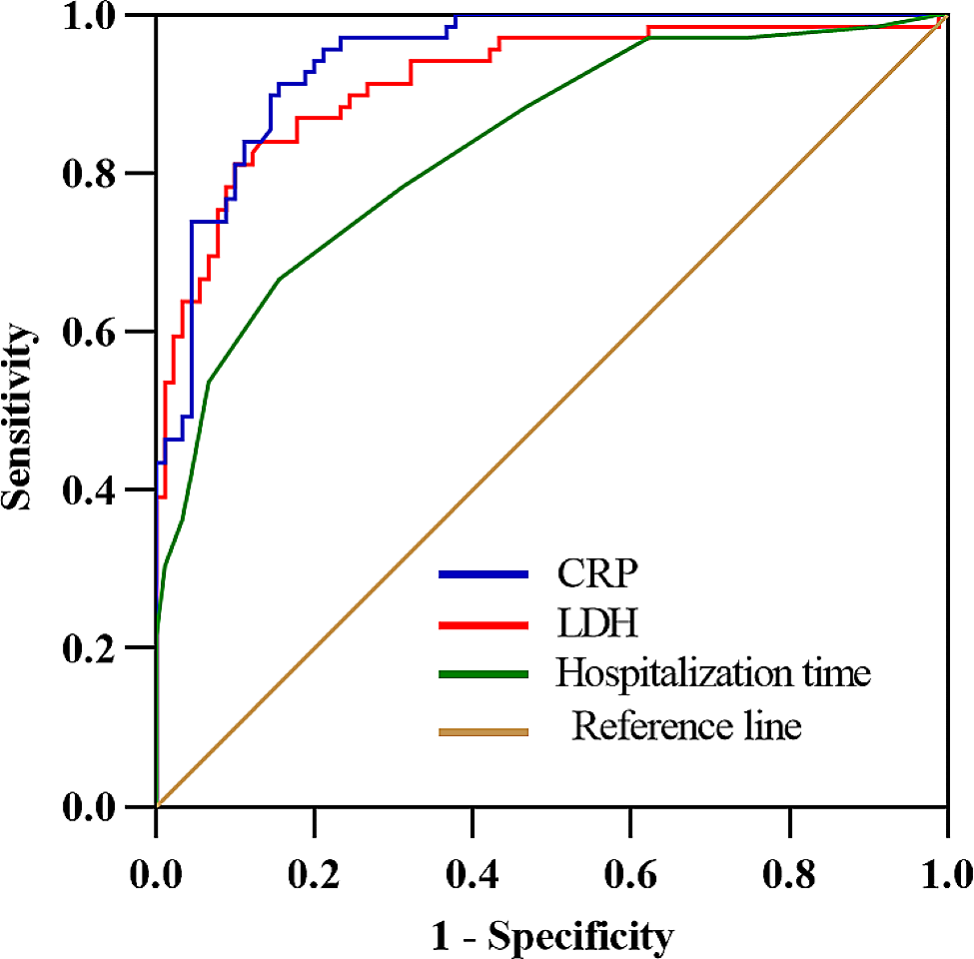
Receiver operating characteristic curve of predictive value of hospital stay, CRP level, and LDH level on delayed radiographic resolution in children with lobar pneumonia induced by *Mycoplasma pneumoniae* CRP, C-reactive protein; LDH, lactate dehydrogenase

The cutoff values for maximum sensitivity and specificity of predictive indicators were determined according to Youden’s method. The results showed that the cutoff values for predictive indicators were a hospitalization duration of ≥ 10.5 days, CRP level of ≥ 25.92 mg/L, and LDH level of ≥ 378 U/L. The corresponding area under the curve was 0.835, 0.944, and 0.917; the sensitivity was 0.667, 0.913, and 0.812; and the specificity was 0.844, 0.844, and 0.900, respectively (Fig. 2; Table 5).

**Table 5:** Receiver operating characteristic threshold values of predictive indexes of delayed radiographic recovery in children with lobar pneumonia induced by Mycoplasma pneumoniae *Mycoplasma pneumoniae*

Variables	Cutoff value	The sensitivity	The specificity	AUC
Hospitalization time (days)	10.50	0.667	0.844	0.835
CRP (mg/L)	25.92	0.913	0.844	0.944
LDH (U/L)	378	0.812	0.900	0.917

CRP, C-reactive protein; LDH, lactate dehydrogenase; AUC, area under the curve.

### Prognosis

Comparing the prognosis of two groups of children, it was found that the patients of DGR had an increased risk of adverse prognosis (*P* < 0.05), such as necrotizing pneumonia, bronchiectasis, obliterative bronchiolitis and recurrent wheezing. The top two are recurrent wheezing and necrotizing pneumonia. They account for 47.8% and 36.2%, respectively (Table 6).

**Table 6:** Comparison of prognosis between NRG and DRG of discharged children

Characteristics	NRG (*n* = 270)	DRG (*n* = 69)	*P*˗value
Necrotizing pneumonia [*n*(%)]	5(1.9)	25(36.2)	< 0.001
Bronchiectasis [*n*(%)]	2(0.7)	10(14.5)	< 0.001
Bronchiolitis obliterans [*n*(%)]	5(1.9)	12(17.4)	< 0.001
Recurrent wheezing [*n*(%)]	11(4.1)	33(47.8)	< 0.001

## Discussion

Lobar pneumonia caused by *M. pneumoniae* in children often presents as pulmonary lobe or segmental consolidation (37%) and slightly less commonly as, infiltration near the hilum or around the bronchus (27%), local reticularis nodule infiltration (21%), and patchy infiltration (15%) [14]. Unilateral pulmonary lobe involvement is more common than bilateral involvement. Imaging examination is one of the main bases for clinical severity and prognosis assessment. In this study, we found that children in the DRG were more likely to have a worsened prognosis. Delayed radiographic resolution may lead to necrotizing pneumonia, bronchiolitis obliterans, bronchiectasis, and recurrent wheezing, their incidence increases in DRG significantly. After the above-mentioned situations occur, almost all patients will experience worsening pulmonary function and decreased exercise tolerance. Therefore, in order to strengthen post-discharge management, early identification of these children is necessary.

This study involved 339 children with MPP who showed lobar changes on lung imaging. The children’s clinical features, laboratory examination findings, and bronchoscopic findings were retrospectively analyzed, and the risk factors for delayed radiographic resolution were summarized. Univariate analysis showed statistically significant differences between the NRG and DRG in the CRP level, LDH level, total fever duration, hospitalization time, glucocorticoid resistance, a large amount of pleural effusion, pulmonary lesions involving two or more lung lobes, and atelectasis (*P* < 0.05). Further analysis using a multivariate logistic regression model and ROC curve showed that a of hospitalization time of ≥ 10.5 days, CRP level of ≥ 25.92 mg/L, LDH level of ≥ 378 U/L, pulmonary lesions involving two or more lung lobes, and a large amount of pleural effusion were risk factors for delayed radiographic resolution of lobar pneumonia caused by *M. pneumoniae* in children. Radiographic resolution of pneumonia is affected by multiple factors, which are generally divided into two categories: factors related to the disease severity and factors related to the treatment effect. The more risk factors are combined in the same children, the greater the probability of delayed radiographic recovery.

More severe clinical features and higher laboratory indicators may be associated with delayed radiographic recovery. Lobar pneumonia caused by *M. pneumoniae* occurs mostly in children aged >5 years, because their immune function is stronger, their autoimmune reaction after *M. pneumoniae* infection is stronger, and lung tissue damage is more serious than in children aged < 5 years. Zhao et al. [15] found that age had an impact on the prognosis of children with MPP, and the critical age threshold was 4.5 years. The mean age of the children in the NRG and DRG in this study was 7.17 ± 2.37 and 6.86 ± 1.50 years respectively, both were above the threshold of 4.5 years, and there was not find any impact of age on the prognosis of the two groups of patients. Patients in the DRG had a longer hospital stay (12.70 vs. 8.36 days) and a longer fever duration (11.13 vs. 6.34 days) than those in the NRG. Moreover, the levels of CRP (57.18 vs. 10.61 mg/L), LDH (515.00 vs. 307.99 U/L), and D-dimer (3.18 vs. 0.85 mg/L) were higher in the DRG than NRG. These findings suggest that the pulmonary inflammatory reaction or tissue damage was more severe in the DRG [16]. An elevated CRP level is an early indicator of lung tissue necrosis, indicating a systemic inflammatory reaction secondary to severe lung infection. Additionally, studies have suggested that the incidence of pleural effusion, myocardial injury, and liver injury is remarkably increased in children who have MPP with elevated indices [17, 18]. Such findings are consistent with the slower radiographic resolution seen in the children in the DRG of our study. The multivariate logistic regression analysis showed that the CRP and LDH levels were independent risk factors for delayed radiographic resolution, and the ROC curve analysis showed that the CRP and LDH levels (area under the curve = 0.944 and 0.917, respectively) had high predictive value for delayed radiographic resolution.

Several serious complications can lead to delayed radiographic resolution of pneumonia. The most common complications of *M. pneumoniae*-induced lobar pneumonia include pleural effusion, atelectasis, and mucus plugs formation, which seriously affect the recovery of lung imaging. Kim et al. [19] showed that children who had MPP with pleural effusion had more severe pneumonia lesions and poorer responses to treatment, resulting in prolonged radiographic resolution of lung abnormalities. In other studies, a large amount of pleural effusion was closely associated with the occurrence of necrotic pneumonia [2, 8, 20]. The imaging changes of necrotic pneumonia were characterized by the emergence of voids after destruction of the lung parenchymal structure, and the radiographic resolution of necrotic pneumonia was thus slower than that of common pneumonia. Undoubtedly, children with extensive pulmonary inflammatory lesions had a longer recovery time. In this study, 70% (235/339) of the children had a single pulmonary lobe lesion, and only 30% of the children had lung lesions involving more than two pulmonary lobes. It indicates that lobar pneumonia caused by *M. pneumoniae* is usually characterized by involvement of a single pulmonary lobe. Notably, however, 72.5% of the children in the DRG had pulmonary lesions involving two or more lobes, suggesting that once inflammatory lesions involve multiple lobes, the recovery period is prolonged. The formation of mucus plugs is an important manifestation of progressive airway mucosal damage, which can lead to irreversible airway dysventilation and atelectasis. Zhang et al. [21] found that children with mucus plugs formation were more likely to have intrapulmonary complications such as pleural effusion and extrapulmonary complications, making treatment more difficult and inflammation more persistent.

Factors related to the treatment effect have slightly different effects on radiographic resolution of pneumonia. Such factors include MAs non-responsiveness, glucocorticoid resistance, and the time to interventional bronchoscopy. Effective antimicrobial and anti-inflammatory therapy helps control disease and shorten the acute phase of disease. MAs therapy is still the first choice for the treatment of *M. pneumoniae* infection, MAs inhibit protein synthesis to achieve an anti-infection effect by binding to domain II and/or V of 23 S rRNA in the ribosomal subunit of 50 S bacteria. In recent years, however, the rate of resistance to MAs has shown an increasing trend. Wang et al. [22] found that 90.94% (1386/1524) of specimens from 1524 children with MPP were resistant to MAs. Gene mutation in the 23 S rRNA domain V of *M. pneumoniae* is the main mechanism of MAs resistance, and A2063 G/C, A2064 G/C, and C2617 G/A have been shown to be mutation sites of MAs resistance [23, 24]. In the study, the rate of MAs non-responsiveness was 38.9% and 50.7% in the NRG and DRG, respectively, with no statistically significant difference between the two groups. This finding indicates that MAs non-responsiveness did not affect the radiographic resolution in children with MPP, which is consistent with the results of reported by Deng et al. [25]. Additionally, Chen et al. [26] stated that MAs resistance may prolong fever duration and treatment time, but the chest X-ray examination findings and laboratory test indicators did not change accordingly. Glucocorticoids are used as immunomodulators to suppress overactive host immune responses, reduce the intensity of local inflammation, and promote recovery from disease. Patients with glucocorticoid resistance often have more severe clinical manifestations and imaging abnormalities. A strong cellular immune response will lead to severe ciliary dysfunction, reduce airway immune function, and destroy ciliary mucus clearance ability, these changes will result in large-scale infiltration, atelectasis, and mucus plugs formation, seriously affecting the recovery of lung inflammation. Bronchoscopy with bronchoalveolar lavage is a rapid and effective treatment for relieving clinical symptoms. Many studies have shown that bronchoscopy with bronchoalveolar lavage is beneficial for removing mucus plugs and has significant advantages for patients with pneumonia characterized by persistent atelectasis [27, 28]. Disease remission has been shown to become greatly accelerated after intervention of bronchoalveolar lavage [29]. The study also showed that the time to interventional bronchoscopy was shorter in NRG than DRG, and early treatment with bronchoalveolar lavage was conducive to the improvement of imaging.

No single clinical or biochemical indicator can accurately predict whether delayed radiographic resolution of pulmonary abnormalities will occur. We performed a multiple logistic regression analysis and ROC curve analysis to eliminate the confounding among study factors and objectively examine the clinical factors influencing delayed radiographic resolution in children with lobar pneumonia caused by *M. pneumoniae*. However, this study also had some limitations; for example, it is a single-center study, some patients were lost to follow-up, and some patients were excluded because of incomplete data or lack of bronchoscopy with bronchoalveolar lavage, which may have biased the results. The sample size should be increased for in further studies. We followed up on the pulmonary function of some patients in the DRG and found that they had varying degrees of decreased pulmonary function. However, pulmonary function tests could not be conducted on young patients (< 6 years old) who were unable to cooperate, preventing an assessment of their pulmonary function. This article also has the shortcoming of not making a comprehensive comparison of pulmonary function.

## Conclusion

In the study, we found that delayed radiographic resolution was mainly associated with the development of complications and high levels of inflammatory markers. Patients with lung lesions involving two or more lobes, a large amount of pleural effusion, high levels of inflammatory markers (CRP level of ≥ 25.92 mg/L, LDH level of ≥ 378 U/L), or a long hospital stay (a hospitalization time of ≥ 10.5 days) had longer radiographic resolution. These five factors are closely related to radiographic resolution, and pediatrician should pay attention to them in our clinical work. These findings may help to identify these patients early in the course of their disease and enhance patient management after discharge.

## Data Availability

Underlying research data and materials can be accessed by contacting the corresponding author.

## Abbreviations

DRG: Delayed recovery group
NRG: Normal recovery group
ROC: Receiver operating characteristic
AUC: Area under the curve
CI: Confidence interval
OR: Odds ratio
MPP: *Mycoplasma Pneumoniae* Pneumonia
MAs: Macrolide antibiotics
WBC: White blood cell count
NE%: Neutrophil ratio
LYM%: Lymphocyte ratio
CRP: C-reactive protein
D-D: D-dimer
LDH: Lactate dehydrogenase
ALP: Alkaline phosphatase
IgA: Immunoglobulin A
IgM: Immunoglobulin M
IgG: Immunoglobulin G
